# Interplay between Mitochondrial Metabolism and Cellular Redox State Dictates Cancer Cell Survival

**DOI:** 10.1155/2021/1341604

**Published:** 2021-11-03

**Authors:** Brittney Joy-Anne Foo, Jie Qing Eu, Jayshree L. Hirpara, Shazib Pervaiz

**Affiliations:** ^1^Department of Physiology, Yong Loo Lin School of Medicine, National University of Singapore (NUS), Singapore, Singapore; ^2^Cancer Science Institute, NUS, Singapore, Singapore; ^3^NUS Center for Cancer Research (N2CR), Yong Loo Lin School of Medicine, NUS, Singapore, Singapore; ^4^NUS Medicine Healthy Longevity Program, Yong Loo Lin School of Medicine, NUS, Singapore, Singapore; ^5^Integrative Sciences and Engineering Program, NUS Graduate School, NUS, Singapore, Singapore; ^6^National University Cancer Institute, National University Health System, Singapore, Singapore; ^7^Faculté de Médicine, Université de Paris, Paris, France

## Abstract

Mitochondria are the main powerhouse of the cell, generating ATP through the tricarboxylic acid cycle (TCA) and oxidative phosphorylation (OXPHOS), which drives myriad cellular processes. In addition to their role in maintaining bioenergetic homeostasis, changes in mitochondrial metabolism, permeability, and morphology are critical in cell fate decisions and determination. Notably, mitochondrial respiration coupled with the passage of electrons through the electron transport chain (ETC) set up a potential source of reactive oxygen species (ROS). While low to moderate increase in intracellular ROS serves as secondary messenger, an overwhelming increase as a result of either increased production and/or deficient antioxidant defenses is detrimental to biomolecules, cells, and tissues. Since ROS and mitochondria both regulate cell fate, attention has been drawn to their involvement in the various processes of carcinogenesis. To that end, the link between a prooxidant milieu and cell survival and proliferation as well as a switch to mitochondrial OXPHOS associated with recalcitrant cancers provide testimony for the remarkable metabolic plasticity as an important hallmark of cancers. In this review, the regulation of cell redox status by mitochondrial metabolism and its implications for cancer cell fate will be discussed followed by the significance of mitochondria-targeted therapies for cancer.

## 1. Introduction

The mitochondrion is a double-membraned organelle that was hypothesized to have evolved from a prokaryote to endosymbionts within eukaryotes [1]. The inner membrane folds on itself to form cristae, enclosing the granular matrix [2]. The significance of the resultant intermembrane space (IMS) is highlighted in its role in the production of cellular energy (ATP) through glycolysis, the tricarboxylic acid (TCA) cycle, and oxidative phosphorylation (OXPHOS). Cancer is characterized by the accumulation of multiple genetic alterations that give rise to multiple mutations, resulting in uncontrolled cell proliferation that requires high energy production and macromolecule synthesis. As such, cancer cells rely on processes such as glycolysis and TCA metabolism for cell survival.

The TCA cycle comprises of a series of biochemical reactions that contribute to energy production and macromolecule synthesis in the mitochondrial matrix. Under normal physiological conditions, the TCA cycle can be divided into two stages. First, citrate is converted into succinyl-CoA through a series of reactions that leads to decarboxylation, releasing two CO_2_ molecules and conversion of NAD^+^ to NADH + H^+^ in the process. Second, succinate is converted to oxaloacetate through successive oxidation steps [3]. Intriguingly, tumor cells have been reported to harbor genetic alterations in enzymes involved in the TCA cycle such as succinate dehydrogenase (SDH) and fumarate hydratase (FH) [4, 5]. Loss of SDH and FH leads to the accumulation of ROS which, in turn, leads to DNA damage and altered cellular processes that contribute to oncogenesis.

Otto Warburg postulated that cancer cells rely on aerobic glycolysis, a term now known as Warburg effect. As such, the role of mitochondrial metabolism in tumorigenesis has been often overlooked until recent years where several evidences point to a reliance on mitochondrial respiration in some cancers. Hence, in this review, we discuss the regulation of mitochondrial metabolism and redox balance in tumorigenesis and evaluate therapeutic strategies designed to target these pathways in cancer.

## 2. Mitochondrial Metabolism Regulates Cellular Redox Status

### 2.1. Mitochondria as a Major Cellular Source of ROS

ROS are molecules that contain oxygen that is derived from incomplete reduction of O_2_. Some ROS molecules include superoxide (O_2_^•-^), hydrogen peroxide (H_2_O_2_), and hydroxyl radical (OH^•^) [6]. ROS production could be attributed to nonmitochondrial ROS-producing enzymes such as NADPH oxidase (NOX) and xanthine oxide (XO) or as a result of electron leakage at the mitochondrial ETC. XO catalyzes the oxidation of xanthine and hypoxanthine in purine metabolism, which leads to the formation of O_2_^•-^ and H_2_O_2._ NOX has also been reported to promote XO-dependent O_2_^•-^ production [7]. NOX family members are transmembrane proteins that generate ROS through the transport of electrons across biological membranes, leading to reduction of oxygen to O_2_^•-^. There are seven isoforms of NOX (NOX1-5, DUOX1-2) that have been identified, each localized in different cell types. For example, NOX1 is commonly found in endothelial cells, neurons, and microglia; NOX2 is found in phagocytes and microvascular endothelial cells while NOX3 is found in renal cells [8, 9]. Interestingly, the interaction between mitochondria and NOX, termed “ROS-induced ROS release” has been highlighted in glucose withdrawal-induced phospho-tyrosine signaling in glioblastoma (GBM) cell lines [10] and is suggested as a mechanism of ROS accumulation to sustain redox activation [11].

The aerobic nature of cellular respiration in the mitochondria makes ROS an inevitable by-product of the redox reactions. The dogmatic view is that excessive production of ROS could be detrimental to subcellular biomolecules, impair cellular processes, and trigger cell damage and death. Electron leakage in the mitochondrial ETC contributes largely to mitochondrial ROS accumulation. Complex I is the largest complex of the ETC and has been observed to be a major source of ROS under pathological conditions. This is due to mutations in the complex I subunits found in approximately 40% of mitochondrial disorders including diabetes and cancer [12, 13]. The Q-cycle is the mechanism of O_2_^•-^ production in complex III, another complex in the ETC. Complex III transfers the electrons from complexes I and II to cytochrome c, and in the process, protons translocate into the inner mitochondrial membrane (IMM). As a result, electrons leak and interact with O_2_, producing O_2_^•-^ in the IMM and mitochondrial matrix [14]. In the cancer context, overexpression of complex III subunits such as UQCR2 and UQCRH has been observed to induce tumorigenesis in colorectal cancer [15], lung carcinoma, and hepatocarcinoma [16] in a ROS-dependent manner. This highlights the role of mitochondrial metabolism in maintaining the redox balance and tumorigenesis.

### 2.2. Cellular Antioxidant Defense Systems

In most cellular processes, homeostatic balance is important in maintaining normal functioning of cells. The production of ROS is countered by antioxidant systems [17], which scavenge harmful ROS that can cause oxidative damage resulting in DNA point mutations [18, 19], disrupted lipid membranes [20], and altered protein function [21–23]. Amongst the many enzymatic antioxidant defenses are the various superoxide dismutase (SOD) and the glutathione (GSH) system. SOD is a family of enzymes that catalyzes the conversion of O_2_^•-^ to H_2_O_2_. In mammals, there are three SODs: cytoplasmic SOD1 (Cu/ZnSOD), extracellular SOD3 (ecSOD) [24, 25], and mitochondrial manganese-dependent SOD2 (MnSOD) [26]. MnSOD is shown to be downregulated in several cancers including lung carcinomas [27, 28]. Intriguingly, MnSOD was observed to be increased in tumor tissues and is associated with drug resistance [29, 30]. Furthermore, MnSOD expression is reportedly increased in aggressive breast cancer and influences epithelial-mesenchymal transition (EMT) in breast cancer [31, 32]. The glutathione peroxidase family (GPx) utilizes reduced GSH as the cofactor in reducing H_2_O_2_ into harmless H_2_O. During the reduction of H_2_O_2_, cofactor GSH is oxidized and disulphide bonded into a GS-SG dimer. Glutathione reductase is then responsible for reducing the GS-SG dimer, replenishing the GSH cofactor for further antioxidant activity [33]. Modified GPx expression has been observed in several cancers such as breast cancer [34], gastric cancer [35, 36], and thyroid cancer [37].

## 3. Dichotomy of Redox Signaling in Cancer Cell Fate Decisions

**Figure 1:**
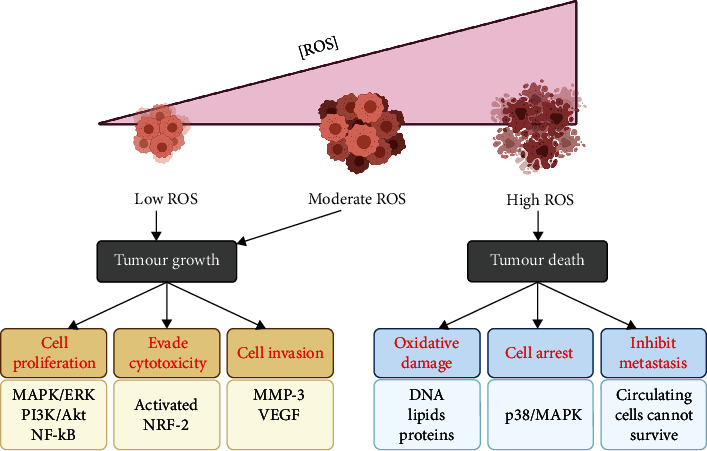
Regulation of carcinogenesis by ROS. At different ROS levels, cells experience a dichotomous fate. Moderate and low ROS levels tend to promote cell growth and uncontrolled proliferation, thus, promoting carcinogenesis and/or its progression. On the other end of the spectrum, high ROS levels tip the balance to cell death, preventing further growth of the tumor. Figure created with biorender.com.

Depending on the type and concentration, ROS can impact cell fate signaling. As a matter of fact, cellular redox status serves as a double-edged sword from the standpoint of carcinogenesis and its progression, as summarized in Figure 1. At moderate but sublethal concentrations, a mild oxidative stress milieu can have prosurvival/proliferation properties. Coupled with the ability of cells to mount sufficiently effective antioxidant defenses, an association between a “pro-oxidant” environment and processes that favor tumor progression such as metastasis has been strongly suggested [38, 39].

### 3.1. Conventional Dogma: ROS are Onco-Suppressors

Conventionally, increased ROS levels have also been implicated in tumor cell growth inhibition. Interestingly, the reactive intermediates damage biomolecules such as membrane lipid bilayer, leading to lipid peroxidation; OH^•^ radical has been shown to attack unsaturated lipids, generating lipid hydroperoxides [40]. A notable by-product of lipid peroxidation is 4-hydroxynonenal (4-HNE) that is found to exert cytotoxic and genotoxic effects [41]. Moreover, low levels of 4-HNE reportedly inhibit c-Myc expression and cell proliferation in leukemia cell lines [42, 43], suggesting an association between ROS-induced lipid peroxidation and inhibition of cancer progression. Furthermore, 4-HNE has been shown to activate both the intrinsic and extrinsic apoptotic pathways [44] as high peroxidation rates overwhelm natural antioxidant systems causing the cells to undergo programmed cell death, thus, highlighting the role of ROS in inhibiting tumor cell growth.

Metastasis can similarly be limited upon exposure to an oxidizing stimulus. Increased oxidative stress inhibits proliferation and survival of circulating cancer cells [45]. In an *in vivo* study conducted by Piskounova et al., NSG mice were subcutaneously transplanted with metastasizing melanoma cells and treated with antioxidant N-acetyl-cysteine (NAC). Interestingly, NAC treated mice were observed to have greater frequency of circulating melanoma cells and increased metastatic disease burden, thereby suggesting that oxidative stress limits the metastatic capacity of melanoma cells [45].

Low dose of ROS activates p53, a tumor suppressor that regulates cellular apoptosis, DNA repair, and cell cycle arrest. Upon ROS activation, p53 in turn downregulates prosurvival proteins such as Bcl-2 and Bcl-xL while activating proapoptotic genes such as Bax, PUMA, and NOXA transcriptionally [46]. Datta et al. showed that in the presence of H_2_O_2_, p53 expression and cell apoptosis were induced. Furthermore, H_2_O_2_-induced apoptosis was abrogated in the presence of p53 antisense oligonucleotides. Similarly, p53-null U373MG cells are resistant to H_2_O_2_-induced apoptosis [47]. The role of p53 in mediating H_2_O_2_-induced apoptosis is supported by Kitamura et al. where they demonstrated an increase in p53 expression as well as Bak, p21WAF1/CIP1 proteins upon H_2_O_2_ treatment [48]. Interestingly, mutant p53 in turn induces ROS accumulation and enhances ROS level through regulation of ROS-related transcription factors such as PGC1-*α* [49, 50]. ROS levels were also seen elevated in p53-induced senescent and apoptotic cells along with decrease in GSH levels in prostate cancer cells [50].

Intriguingly, ROS has been implicated in senescence-induced tumor suppression in several cellular senescence studies. It is noteworthy that ROS-induced oxidative damage is a signal for irreversible cell cycle arrest or senescence [51]. In particular, telomeres are observed to be sensitive to increase in ROS [52], supporting the view that mtROS results in telomere dysfunction and therefore premature senescence [53]. ROS can also serve as secondary messengers in senescence-inducing pathways such as p53/p21^WAF1^ [54], p16^INK-4a^ [55], and p38^MAPK^ [56, 57]. Senescence is closely associated with p16^INK-4a^, and Takahashi et al. demonstrated that ROS determines irreversibility of senescence-induced cell cycle arrest and treatment with NACrescued cells and reinitiates cell proliferation, suggesting a role of ROS in regulating tumor suppression through senescence [58].

### 3.2. Flip Side of the Coin: ROS are Oncogenic

Interestingly, while ROS has been shown to affect cell fate and signaling in cancer, in a rapidly proliferating cell, such as a cancer cell, increased metabolic activity in turn leads to high levels of ROS.

#### 3.2.1. ROS Regulation of Keap1/Nrf2 Complex

As a protective measure, reinforcement of antioxidant defenses such as the Nrf2 pathway provides cells with the ability to adapt, thereby evading oxidative stress-mediated cytotoxicity and tissue damage. Nrf2 is a transcription factor that controls nuclear antioxidant response elements (ARE) in the promoter region of target genes [59], such as those utilized by oncogenes K-Ras^G12D^ and B-Raf^V619E^ in human pancreatic cancer cells *in vivo* [60] and *in vitro* [61]. Further supporting that, Nrf2-knockout mice exhibit increased oxidative stress and predilection for carcinogenesis [62, 63]. Aside from the direct effect of Nrf2 on antioxidant defense reinforcement, increased oxidative stress also promotes stability of Nrf2 via oxidative modification of its regulator Keap1, which promotes Nrf2 proteasomal degradation by polyubiquitination [64]. Keap1 has redox-sensitive cysteine thiols that are prone to modification by H_2_O_2_, altering the Keap1/Nrf2 complex and ultimately inhibiting Nrf2 degradation [65]. Other antioxidant systems like glutathione and thioredoxin have also been shown to promote breast tumor progression in a synergistic manner [66]. However, in other cancer types such as lung [67] and prostate [68], SOD2 levels have been found suppressed, hence, suggesting that the regulation of intracellular antioxidants depends on the primary tumor.

#### 3.2.2. Oncogenic Mutations Tilt the Balance of ROS

ROS levels could also be affected by oncogene mutations arising from chromosomal translocations. The fusion protein BCR/ABL in the Philadelphia chromosome is characteristic of predominantly chronic myeloid leukemia (CML) and acute lymphoblastic leukemia (ALL) [69]. This hybrid protein has constitutive tyrosine kinase signaling properties that allow uncontrolled cell cycle progression [70]. Increased H_2_O_2_ levels have been found in BCR/ABL-activated cell lines [71–73], followed by a decrease of BCR/ABL activity in the presence of antioxidants [73]. Additionally, ROS from BCR/ABL activation can further mutate the fusion BCR/ABL gene to confer therapeutic resistance against specific tyrosine kinase inhibitors [74]. Another chromosomal translocation is NPM/ALK, which also displays upregulated tyrosine kinase activity in anaplastic large-cell lymphoma (ALCL) [75]. The mutation leads to downstream ROS production via the lipoxygenase enzyme family [76], where ROS can act as secondary messengers to activate pathways implicated in tumorigenesis such as MAPK for metastasis [77].

#### 3.2.3. ROS-Mediated Genome Instability

As genomic instability is a hallmark of cancer [78], ROS-mediated DNA damage hints to cancer formation. Under oxidative stress, ROS oxidizes DNA bases. The most common oxidation is guanine to 8-oxo-dG due to it having the lowest reduction potential amongst the other bases [79–82]. 8-oxo-dG is a DNA lesion and can lead to permanent mutations which modify gene expression. In 1990, the association between 8-oxo-dG and carcinogenesis was established [83] although the direct link has not been found. Notably, DNA changes such as base substitutions occur in cells with artificially added 8-oxo-dG [84]. A common mutation is the transversion of oxidized guanine to thymine [85, 86]. As a matter of fact, 8-oxo-dG has been proposed as a marker of neoplastic transformation, thus, linking oxidation-induced DNA damage to carcinogenesis [87].

Genomic instability is promoted by the loss of tumor suppressors that control cell cycle progression and DNA damage repair process, such as the master transcription factor, p53, which is mutated in more than 50% of human solid tumors [88, 89]. At moderate sublethal ROS, p53 upregulates antioxidants to evade oxidative stress [90–92]; however, in the face of cytotoxic ROS levels, p53 downregulates antioxidants to tip the cell towards apoptosis [93, 94]. As such, the dual role of p53 in regulating cellular redox state switches the pendulum from survival to death execution [95]. Mechanistically, loss-of-functional of p53 increases ROS levels via the TP53-inducible glycolysis and apoptosis regulator (TIGAR). TIGAR normally shifts carbon from glycolysis to the pentose phosphate pathway (PPP) by degrading the allosteric activator of phosphofructosekinase-1 (PFK1) and NADPH generated from PPP can fuel antioxidant processes by glutathione [92, 96]. Reduced GSH can then convert H_2_O_2_ to water, reducing ROS levels. Without functional p53, ROS levels are no longer regulated by the TIGAR-dependent process. Besides the downstream ROS effects of p53, it is worthy to mention that p53 itself contains conserved cysteine residues that are susceptible to redox modification [97].

#### 3.2.4. Effect of ROS on Signaling Pathways

**Figure 2:**
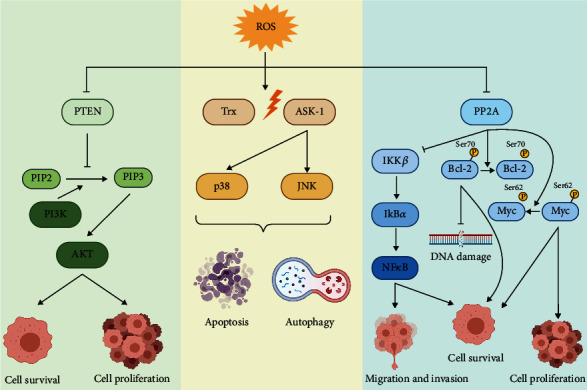
ROS regulation of signaling pathways in cancer. ROS has been demonstrated to regulate signaling pathways in cancer. Increase in ROS such as H_2_O_2_ activates PI3K/Akt signaling pathway through the inhibition of PTEN, resulting in constitutive activation of PI3K/Akt that contributes to cell survival and proliferation in cancer. Similarly, ASK-1, a kinase in the MAPK signaling cascade, is displaced from Trx-ASK1 complex under oxidative stress. ASK-1 subsequently activates downstream p38 and JNK, leading to apoptosis and autophagy. Redox-mediated inactivation of PP2A was reported to sustain NF-*κ*B activation, Bcl-2 phosphorylation at Ser70, and Myc phosphorylation at Ser62, leading to cell migration and invasion, inhibition of DNA damage and cell proliferation, respectively. Figure created with biorender.com.

ROS can also damage functional proteins directly by oxidizing susceptible catalytic thiol groups in enzymes [19, 22, 98]. In this regard, ROS can activate signaling pathways that promote cell proliferation and survival as a secondary messenger [99]. A large body of evidence appears to implicate intracellular H_2_O_2_, due to its diffusion efficiency and ubiquitous nature in most cells, as the major ROS involved in most physiological redox signaling [100]. The mechanism of ROS signaling is via its oxidation of thiol “switches” [101] in redox-sensitive substrates that have conserved cysteine residues [102, 103]. To that end, increased ROS has been shown to activate PI3K/Akt pathway to drive cell survival via inactivation of its regulator protein, phosphatase and tensin homolog (PTEN) [22, 104]; exogenously added H_2_O_2_ can directly oxidize cysteine residues on PTEN, thereby compromising its PI3K/Akt-regulating activity [19, 22, 105] (Figure 2). Interestingly, constitutively activated PI3K/Akt pathway results also results in an increase in intracellular O_2_^•-^ production as a side-product from the generation of prostaglandin by COX enzymes with peroxidase activity [106, 107]. The involvement of NOX family of oxidase has also been shown in Akt-induced O_2_^•-^ production [108], which has been associated with mutation(s) of RAS, an oncogene that is constitutively activated in approximately 20-30% of human cancers [109]. RAS is a GTPase protein that is activated downstream of growth factor receptor tyrosine kinase (e.g., EGFR) which then activates PI3K by phosphorylation. Although NOX-mediated ROS production is mainly recognized for phagocytic respiratory burst, and its significance in tumor angiogenesis and metastasis is not dismissed [110]. Direct pathway components can also increase ROS levels, such as the catalytic subunit of PI3K (encoded by *PIK3CA*), which are found to be mutated in most solid tumors [111–113]. Mutated PIK3CA cell lines were observed to display raised *α*-KGDH activity, a source of ROS [114]. Further corroborating this, negative regulators of the PI3K pathway, such as PTEN, are tumor suppressors that can be modulated by ROS.

Similarly, signaling pathways like the mitogen-activated protein kinase (MAPK/ERK) have demonstrated mediation by ROS, and other studies conversely demonstrate the ability of antioxidants to alleviate MAPK activation [77]. Oxidative stress upon exposure to exogenously added H_2_O_2_ also correlated with MAPK activation [115, 116]. For example, apoptosis signal-regulating kinase-1 (ASK-1), a kinase in the MAPK signaling cascade, is displaced from antioxidant protein, thioredoxin, under oxidative stress, and oligomerizes to activate downstream p38 and c-jun N-terminal kinase (JNK) [117]. Moreover, ASK-1 expression has been associated with gastric cancer progression, where cell proliferation decreased in ASK-1 knockdown gastric cancer cells [118]. Since oxidative stress is reportedly the most common activator of ASK-1, this highlights the prominent role of oxidative stress in carcinogenesis through the regulation of signaling pathways (Figure 2).

#### 3.2.5. Oxidative Stress-induced Metastasis, Angiogenesis and Cell Death Inhibition

Oxidative stress is also able to drive hallmarks involving cell motility and invasion such as metastasis and angiogenesis. Kundu et al. demonstrated that exposure to sublethal exogenous H_2_O_2_ in murine cancer cells increased rates of metastasis and anchorage-independent survival while reducing tumor cell adhesion to ECM [119]. H_2_O_2_ has been shown to regulate the expression of matrix metalloproteases (MMPs) that disrupt the ECM to facilitate cancer cell invasion [120]. Along similar lines, MMP-3 was shown to increase oxidative stress and induce EMT in murine mammary epithelial cells [121]. Moreover, H_2_O_2_-associated EGFR signaling is also vital for tumor metastasis [122], where murine metastatic melanoma colonies had greater EGFR expression [123]. Oxidative stress has been shown to stabilize HIF-1*α* and allow the initiation of VEGF expression, which leads to the induction of angiogenesis [124], thus, supplying oxygen and nutrients to hypoxic tumors [125]. Furthermore, the NF-*κ*B pathway associated with processes involved in carcinogenesis such as inflammation, cell survival, migration, and invasion is activated by H_2_O_2_ via IKK-dependent mechanism [126] as well as by redox-mediated inactivation of the phosphatase PP2A [127], which regulates phosphorylation-dependent degradation of I*κ*B*α*, further corroborating the involvement of ROS in processes associated with metastasis. Likewise, redox-mediated inactivation of PP2A has also been associated with phospho-stability of the antiapoptotic protein Bcl-2 (pS70) and oncogene c-Myc (pS62), specifically implicating O_2_^•-^-mediated ONOO^−^ induced tyrosine nitration [128, 129]. Sustained pS70 of Bcl-2 in turn blocks oxidative stress-induced DNA damage to promote cancer cell survival [130]. Similarly, death receptor inhibitory protein, cFLIP, was shown to be upregulated upon an increase in intracellular O_2_^•-^, thereby blunting death receptor signaling [131].

## 4. Redox Dysregulation and Mitochondrial Metabolism in Cancer

### 4.1. Warburg and Reverse Warburg Effects

Mitochondrial ROS can impact mitochondrial function, which can ignite further imbalance in redox homeostasis. The main mitochondrial function is the metabolism of organic substrates such as glucose, lipids, amino acids, and nucleic acids. In terms of energy production from cellular respiration, the metabolism of glucose for ATP has been observed to be dysregulated in cancer cells that are actively proliferating. As mentioned earlier, the Warburg effect postulated that cancer cells rely on aerobic glycolysis to rapidly produce energy for proliferation due to mitochondria dysfunction and suppression of OXPHOS. Pyruvate is an end-product of glycolysis that exerts antioxidant effect and protects the mitochondria from oxidative damage [132]. Wang et al. reported that pyruvate is able to inhibit O_2_^•-^ production in the presence of mitochondrial complex inhibitors and inhibits mitochondrial ROS generation to a greater extent compared to intracellular ROS levels [132]. Moreover, it was observed that intracellular increase in ROS levels leads to inhibition of pyruvate kinase M2 isoform (PKM2), a glycolytic enzyme that plays a vital role in catalyzing the conversion of phosphoenolpyruvate to pyruvate [133]. PKM2 has also been reported to serve as a metabolic sensor under glucose-starved conditions, as inhibition of PKM2 was observed to enhance metabolic activity and protect against apoptotic cell death [134].

However, this milestone discovery is challenged by studies demonstrating that glycolysis accounted for less than half of the ATP produced in multiple malignant cell lines [135]; OXPHOS still contributed the majority of ATP during normoxia and lesser than 50% during hypoxia in malignant breast and cervical cell lines [136, 137]. The effectively coined “*Reverse Warburg Effect*” has brought attention to the tumor microenvironment (TME), where stromal cells such as cancer-associated fibroblasts (CAFs) could be responsible for allowing malignant cells to produce ATP while reducing reliance on OXPHOS [138, 139]. Oxygen becomes a limiting factor when proliferation is uncontrolled, producing ROS such as H_2_O_2_ to initiate CAF production of high energy metabolites such as pyruvate and lactate. These products are then transported to the tumor cells to be funneled into OXPHOS to produce a significant amount of ATP [140], demonstrating altered metabolism within tumor mitochondria. In fact, OXPHOS has been shown to be upregulated in ovarian cancer stem cells [141] as well as drug-resistant cancers [142–144] which give rise to an emerging number of studies on targeting OXPHOS in cancer therapy.

### 4.2. ROS and Nuclear-Encoded Mitochondrial Proteins

**Figure 3:**
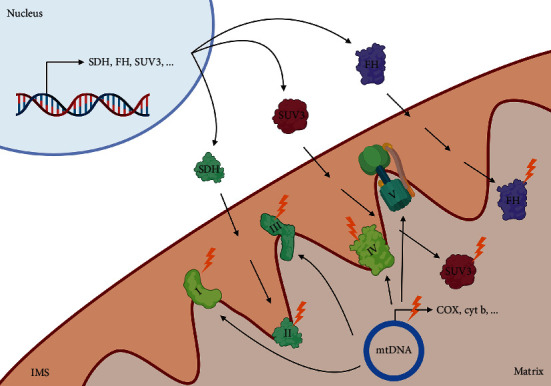
Nuclear-encoded and mitochondrial-encoded mitochondria regulatory genes are susceptible to oxidative damage. Nuclear-encoded mitochondrial genes such as SDH, which makes up complex II of the ETC, are assembled and transported into the mitochondria and susceptible to inactivation by ROS such as H_2_O_2_. In addition, FH generates energy for the cells by converting fumarate into malate in the TCA cycle, and SUV3 is a nuclear-encoded ATP-dependent DNA/RNA helicase and function as a tumor suppressor. The mtDNA encodes for subunits of the ETC and regulatory proteins that are important for the assembly and function of the ETC. The mtDNA is highly susceptible to oxidative stress-induced damage, leading to OXPHOS dysregulation. Figure created with biorender.com.

Nuclear-encoded mitochondria genes are susceptible to oxidative damage (Figure 3). Some of the protein products of nuclear-encoded mitochondrial genes include *SDH* [4, 5] and *FH* [5]. Inactivation of these enzymes by H_2_O_2_ [145] or by somatic mutations in the enzymatic subunits [5] could reduce the rate of ATP and ROS production during OXPHOS. The inactivation of these enzymes could trigger cellular transformation, as evidenced by the association of *SDH* subunit mutations with paraganglioma [146, 147]. SDH, also known as complex II of the mitochondrial ETC, is an important ROS-producing site, which either generates ROS directly from the release of electrons from the conversion of FAD to FADH_2_ under low succinate concentration condition, or indirectly through reverse electron transfer (RET) in the presence of high concentration of succinate, forcing electrons through complex I [148]. Loss of function mutations in *SDH* reported in HPGL/PCC cancer could lead to an accumulation of succinate and ROS which leads to further oxidative stress and DNA hypermutations [149].

Similarly, altered FH activity has been implicated in renal cell carcinoma and uterine leiomyoma [150–152]. Patients with germline mutations in *FH* were also observed to have a higher risk in cancers such as renal cell cancer, breast, and bladder cancer [134]. As FH catalyzes the conversion of fumarate to malate, loss-of-function mutations of *FH* lead to fumarate build up, which can activate Nrf2 by inhibiting Keap1 [153]. The protective effect of the genes regulated by Nrf2 downstream of the ARE, such as heme oxygenase 1 (*HMOX1*), promote tumorigenesis by alleviating oxidative stress [154, 155].

In addition, nuclear-encoded *Suppressor of Var1* (*SUV3*) RNA helicase is responsible for mitochondria DNA (mtDNA) replication and murine haploinsufficiency of *SUV3* allele predisposes to tumorigenesis *in vivo* [156], indicating its role as a tumor suppressor. Furthermore, complete knockdown of *SUV3* results in a decrease in mtDNA copy number and, subsequently, together with an increase in O_2_^•-^ formation and enhanced tumorigenesis [157]. *SUV3* knockdown also observed change in mitochondrial morphology and eventual senescence [156].

### 4.3. ROS and Mitochondria-Encoded Proteins

Since mitochondria are an important source of ROS, mtDNA is highly vulnerable to oxidative stress-induced damage in the prooxidant milieu of cancer cells, as mtDNA is not protected by histone proteins [158]. The mitochondrial genome encodes for 13 OXPHOS subunits, 22 tRNAs, and 2 rRNAs [159], including cytochrome proteins that are critical for optimal functioning of the ETC (Figure 3).

#### 4.3.1. Cytochrome c Oxidase (COX)

In particular, mitochondria-encoded COX displays antioxidant activity to lower ROS levels, which prevents further oxidative damage [160]. Furthermore, studies have also reported a decrease in COX activity and expression, associated with increased ROS production in human colon adenocarcinoma [161, 162] and murine hepatoma cells [163].

#### 4.3.2. Cytochrome b

MtDNA also codes for *cytochrome b*, which is the only component of complex III encoded by the mtDNA. Cytochrome b is found to be mutated in bladder cancer, which leads to increased ROS production coupled with amplified NF-*κ*B signaling and tumor cell growth. Overexpression of *mtCYB* was associated with increased tumor growth and invasion *in vivo.* Interestingly, inhibition of ROS inhibited cell proliferation driven by NF-*κ*B, thus, suggesting that increase in ROS upon *cytochrome b* mutation is involved in mediating cell proliferation in bladder cancer [164].

#### 4.3.3. Mitochondrial-Encoded Complex I

The mtDNA codes for 7 subunits of the complex I—ND1, ND2, ND3, ND4, ND4L, ND5, and ND6. Mutations in mtDNA most commonly affect complex I genes and induce feedback induction of Warburg effect and AMPK activation. Iommarini et al. also demonstrated that different degrees of complex I dysfunction induced differential oxidative stress. Severe mutations lead to complex I disassembly, inhibiting the transfer of electrons to ROS-generating complexes leading to inhibition of ROS generation [165].

#### 4.3.4. ATP Synthase

mtDNA codes for 2 subunits of ATP synthase—MT-ATP6 and MT-ATP8. Mutations occur at two amino acid positions 8993T>G and 8993T>C, which has reportedly caused 90% and 70% deficit, respectively, in ATP synthase function due to inefficient assembly and stability of the subunit [166]. Mutations in ATP6 and ATP8 genes were observed in breast cancer although functional effect of the mutation has yet to be studied widely in the model [167].

### 4.4. Effect of Aberrant ROS Signaling on Mitochondrial Apoptotic Pathway

Altered redox metabolism also regulates mitochondrial (intrinsic) apoptotic signaling. Singh et al. demonstrated that H_2_O_2_ induces Bax expression with a reciprocal decrease in antiapoptotic protein Bcl-xL in HeLa cells, which triggered cytochrome c release from mitochondria [168]. This is supported by several studies demonstrating the importance of H_2_O_2_ as a signaling molecule for apoptosis induction [169–171]. On the other hand, H_2_O_2_ was observed to inhibit drug-induced apoptosis through the depletion of cellular energy (ATP) by activation of PARP [172, 173], hence, suggesting a dual role of H_2_O_2_ in regulating apoptotic signaling. Other redox-sensitive proteins such as VDAC and ANT can stimulate MOMP and cytochrome c release upon exposure to H_2_O_2_ or O_2_^•-^[169, 174, 175]. Furthermore, increased H_2_O_2_ levels coincide with increased FADD intermembrane translocation and FasL, which activate initiator caspase-8 in the extrinsic apoptotic pathway, whereas decreasing O_2_^•-^ sensitizes Bcl-2 overexpressing cancer cells to receptor or drug-induced apoptosis [176, 177]. Collectively, these findings highlight the critical role of an altered redox state in mitochondria-dependent apoptotic execution.

## 5. Targeting Mitochondria as a Therapeutic Strategy

**Figure 4:**
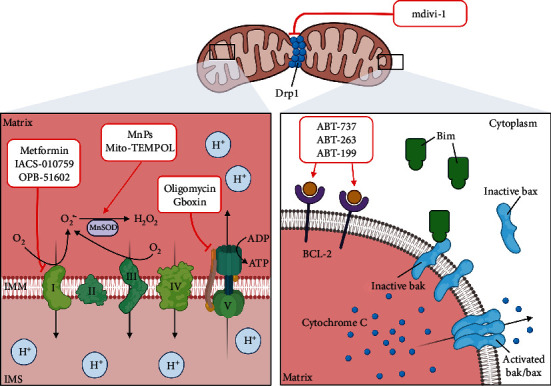
Mitochondrial-directed therapeutic strategies. For drugs that target the mitochondrial matrix, components of the electron transport chain and MnSOD are mainly affected, whereas drugs targeting cytosolic regulatory proteins/factors such as the balance between the pro- and antiapoptotic members of the Bcl-2 family affect apoptotic execution. Figure created with biorender.com.

Since cancer cells harness ROS at a level that stimulates proliferative and survival signaling without being cytotoxic, therapeutics aim to create oxidative stress to drive transformed cells towards apoptotic clearance. Here, we compare several compounds developed to target mitochondria and ROS production in cancer (Table 1, Figure 4). The oxidative burden generated can also confer sensitivity to other anticancer drugs.

### 5.1. Complex I Inhibitors

Although metformin was developed as a diabetic drug, there is an increasing interest in its anticancer properties through inhibition of complex I of the ETC, which disrupts ATP production by OXPHOS. Metformin was also reported to indirectly reduce mitogenic insulin growth factors when controlling blood glucose and preventing PI3K activation of cell proliferation [178]. Metformin exerts antioxidant properties through inhibition of protein kinase C activity and, in turn, leading to decreased ROS production [179]. The clinical efficacy was reflected in the phase III randomized trial breast cancer patients where the compound displayed increased progression-free survival (NCT01101438). In addition, the efficacy of metformin in combination with chemotherapy such as gemcitabine as well as targeted therapies such as erlotinib such as tyrosine kinase inhibitors (TKI) has been evaluated in phase II clinical trials in pancreatic cancer patients (NCT01210911), although more studies need to be conducted to evaluate the safety and efficacy of combination therapies with metformin.

Small molecule inhibitor IACS-010759 also attacks complex I, displaying *in vitro* benefits in both AML and CLL. AML cells were sensitive to IACS-010759 treatment where cell viability and oxygen consumption rate were decreased [180]. In CLL, tumor cells exposed to IACS-010759 had lower OXPHOS rates but adapted by relying on glycolysis for survival, highlighting that maximum IACS efficacy can be achieved by inhibiting both glycolysis and OXPHOS [181]. A recent phase I trial also pointed out the antitumor potential of IACS-010759 in pancreatic cancer, triple-negative breast cancer, and SWI/SNF-related tumors (NCT03291938) [182]. Combination therapy of complex I inhibitor IACs-010759 with vinorelbine was shown to have a synergistic effect in primary cells of AML patients [183]. Currently, IACS-010759 is also in phase I study in relapsed/refractory AML (NCT02882321) and variety of solid tumors (NCT03291938) as well as BCL2 inhibitor venetoclax (ABT-199). Dual therapy of IACS-010759 with venetoclax has shown elimination of leukemic cells in *in vitro* and *in vivo* AML models by inhibiting mitochondrial respiration and BCL2/VDAC interaction [184]. IACS-010759 has also shown prolonged survival effect in PD-1 resistant NSCLC in combination with radiation therapy and anti-PD1 by inhibiting RT-induced immunosuppression [185].

OPB-51602 (OPB) is another novel complex I inhibitor that has shown high specificity and produced antitumor effects in drug-resistant cancer cell lines [143]. A previous phase I trial on OPB's clinical effectiveness showcased preliminary benefits of OPB in TKI-resistant cancers [186]. Although known to restrict respiration by interfering with STAT3 signaling [187], Hirpara et al. have demonstrated that OPB specifically inhibits complex I due to increase in O_2_^•-^ production and reduction in NAD^+^/NADH ratio, an indicator of complex I inhibition. Furthermore, OPB proved effective in patients with TKI-resistant EGFR mutation NSCLC, by reducing tumor burden significantly [143]. OPB was also shown to have significant sensitizing effect with TKI in variety of cancer cells [186].

### 5.2. ATP Synthase Inhibitor

The final OXPHOS step can also be targeted by ATP synthase inhibitors such as oligomycin [188]. The disruption of ATP production similarly causes O_2_^•-^ formation from resultant electron leaks in the ETC. Oligomycin is found to specifically inhibit membrane-bound F_O_ region of ATPase [189]. Recent studies demonstrate how oligomycin helps resensitize leukemic cells to TKI treatment [190]. The treated leukemic cells were subsequently responsive to Bcr/Abl inhibition, where they had decreased ATP production and increased superoxide production and apoptosis occurrences [190].

A novel ATP synthase inhibitor that has similar effects to oligomycin is positively charged Gboxin, which interacts with the ETC complexes I, II, IV, and ATP synthase to restrict ATP synthase activity in glioblastoma (GBM) [191]. Gboxin-sensitive GBM cells lacked expression of mitochondrial permeability transition pore (mPTP) that could prevent ROS accumulation in the mitochondria, hence, making them susceptible to Gboxin. Gboxin significantly increased mitochondrial membrane potential, with contrast to complex I and III poisons (rotenone and antimycin respectively) which reduced the membrane potential [191]. Gboxin analogue (S-Gboxin) for *in vivo* studies also demonstrated inhibited GBM growth, indicating promising antitumor potential [191].

### 5.3. Mitochondria Biogenesis Targeting Compounds

On top of direct inhibition of the mitochondrial ETC complex activity, there is an emerging interest in targeting mitochondria biogenesis, which is a process defined by an increase in mitochondria mass through the increase in size and number of mitochondria in cells. Mitochondria alleviate oxidative stress through the regulation of several processes including the biogenesis of new mitochondria and mitochondria fusion/fission processes [192]. Although mitochondria biogenesis is regulated by several key factors including PPAR gamma coactivator-1*α* (PGC-1*α*), Nrf1, Nrf2, and mitochondria transcription factor A (TFAM), direct inhibitors to these regulators have yet to be developed. Nevertheless, several drugs have been reported to show a direct impact on mitochondria biogenesis.

Gamitrinib is a small molecule mitochondrial HSP90 inhibitor that has shown promising antitumor effects in various cancer types including glioblastoma [193, 194] and prostate cancer [195]. Unlike other general HSP90 antagonists, gamitrinib selectively targets tumor mitochondria and does not affect the HSP90 homeostasis in other cellular compartments other than the mitochondria and does not induce toxicity to normal cells. Gamitrinib was observed to induce mitochondria apoptosis and a loss of inner membrane potential, which leads to the release of mitochondrial cytochrome c [196]. Gamitrinib has also been reported to lead to a decrease in mtDNA copy number, mitochondria mass, and respiration in melanoma cells, which is indicative of an inhibition of mitochondria biogenesis. Pretreatment with gamitrinib was able to sensitize vemurafenib-resistant A375 cells to vemurafenib treatment through inhibition of mitochondria biogenesis and activity [197].

Doxycycline is an FDA-approved antibiotic used in the treatment of various infections through targeting bacterial mitochondria. In cancer, doxycycline was observed to limit self-renewal ability of cancer stem cells (CSCs) in several types of cancers [198]. In addition to bacterial mitochondria, doxycycline was shown to inhibit mitochondrial biogenesis in mammalian cells; doxycycline treatment decreased mtDNA copy number and mitochondrial translation, eventually leading to cell death [199].

### 5.4. Inhibition of Mitochondria Dynamics

Mitochondria dynamics refer to the balance between fusion and fission that maintain mitochondria morphology and number in mammalian cells. Mitochondria fission is elevated under stress conditions, which induces mitochondrial fragmentation [200]. Impairment of mitochondria fusion and fission results in structural changes, cellular dysfunction, and damage [201]. Mitochondrial division inhibitor (mdivi-1) was reported to inhibit Drp1-dependent mitochondria fission and oxidative metabolism and impair cell proliferation in lung cancer [202]. Mdivi-1 was also reported to inhibit complex I function and modulate intracellular Ca^2+^ signaling [203]. The inhibition of mitochondria fission by mdivi-1 reduces tumor growth through prevention of cell cycle progression [204] and cell migration [205], suggesting mitochondrial dynamics as one of the targets and its inhibition as a way to suppress cancer cell growth.

### 5.5. SOD Mimetics

MnSOD (SOD2) is shown to be frequently dysregulated in several cancers, and thus, there is an emerging interest in complexes that mimic MnSOD (MnSOD mimetics) as therapeutic agents against cancers with suppressed MnSOD activity. SOD mimetics are modulators of cellular redox environment, and several compounds have been examined and reviewed in detail by Vincent et al., Batinic-Haberle et al., and Miriyala et al. [206–208]. In this section, we aim to evaluate the reported antitumor properties of some promising therapeutic MnSOD mimetics.

Manganese porphyrins (MnP) are Mn-based SOD mimetics and have been identified as potential therapeutics due to their high SOD-like activity. In addition, MnP have been shown to regulate signaling pathways, which in turn modulate cellular processes such as apoptosis and proliferation. With excellent bioavailability and specificity for targeting mitochondria, MnTnHex-2-PyP^5+^ is considered one of the most promising therapeutic SOD mimics. Treatment with MnTnHex-2-PyP^5+^ increases cellular ROS levels and induces cell death in human renal cancer cells, 786-O [209]. Furthermore, MnTnHex-2-PyP^5+^ was shown to inhibit cell migration, chemotaxis, and invasion in doxorubicin-treated breast cancer, demonstrating therapeutic potential in several cancer models [210]. Interestingly, another MnSOD mimic, MnTnBuOE-2-PyP^5+^ (MnBuOE/BMX-001) has been reported to enhance carbenoxolone- (CBX-) mediated tumor necrosis factor-related apoptosis-inducing ligand- (TRAIL-) induced apoptosis in GBM cells. MnBuOE also demonstrated an enhanced mitochondrial over cytosolic accumulation [211]. BMX-001 is currently in two Phase II studies—one in combination with radiation therapy and tamazolamide in glioblastoma and high-grade glioma and another in patients with multiple brain metastases (MBM) undergoing whole brain radiation therapy. In addition, the safety and tolerability of BMX-001 are currently being evaluated in two Phase I/II studies using a combination approach of BMX-001, standard radiation therapy (RT), and cisplatin in head and neck cancer patients to reduce radiation-induced mucositis and xerostomia, and in combination with 5-FU in anal squamous cell carcinoma (ASCC) (ClinicalTrials: NCT02655601, NCT02990468, NCT03386500, NCT03608020) [212].

Nitroxides are weak SOD mimetics which have been used as an antioxidant both *in vivo* and *in vitro.* Mito-TEMPOL is a mitochondria-targeted derivative of TEMPOL, which catalyzes the dismutation of O_2_^•-^[213]. Mito-TEMPOL possesses a triphenylphosphonium group which enhances its accumulation in the mitochondria. In a study by Dickey et al., Mito-TEMPOL treatment led to a reduction in tumor size as a single agent and enhanced the antitumor effect of doxorubicin in a murine syngeneic breast cancer model. Furthermore, Mito-TEMPOL was also observed to induce DNA damage, apoptosis, and mitochondrial distress in the tumors [214], highlighting its potential as a therapeutic antioxidant in cancer.

### 5.6. Bcl-2 Inhibitors

Antiapoptotic proteins like Bcl-2 are implicated in the intrinsic apoptotic pathway. The proteins prevent the oligomerization of effector Bax/Bak proteins into a membrane pore, disallowing the release of cytochrome c [215] and modulate ROS production through regulation of mitochondria respiration [128, 216]. Cytochrome c is responsible for the activation of downstream caspase 3 and 8 which result in apoptosis [217]. BH3-only proteins (e.g., Bim, tBid, and PUMA) naturally induce apoptosis by inhibiting Bcl-2 proteins directly, facilitating MOMP for cytochrome c release [218]. The role that Bcl-2 plays in apoptosis makes it an attractive drug target for novel inhibitors that aim to induce apoptosis in tumor cells. BH3 mimetics such as ABT-263 (Navitoclax) and ABT-737 targets both the main antiapoptotic proteins, Bcl-2 and Bcl-xL [219]. ABT-737 displayed antitumor potential in animal hematological cancer models, while a more stable ABT-263 has been tested in phase I and II trials for human hematological cancers too [220].

However, thrombocytopenia is a significant toxicity that challenges the effectiveness of ABT-263 and ABT-737 [220, 221]. To combat this toxicity, a recently approved BH3 mimetic called ABT-199 (venetoclax) is proving to be specific for Bcl-2, possessing subnanomolecular affinity for Bcl-2, while not targeting Bcl-xL [222]. ABT-199 was shown to display a significant response rate in a phase I trial with CLL patients, with common side effects like diarrhea and neutropenia [223]. Phase II trials for ABT-199 on high-risk relapsed and refractory AML patients demonstrated promising clinical benefits with tolerable safety profile [224]. ABT-199 was also effective in acute leukemia for both in vitro [225] and in vivo mouse studies [226]. BH3 mimetics displace BH3-only sensitizers such as Bim from binding with Bcl-2, directly inhibiting Bcl-2 and allowing free Bim to activate Bax/Bak for cytochrome release [227, 228]. However, a recent publication posits that BH3-only proteins can interact with Bax/Bak but is not needed for apoptosis [229], which is consistent with an alternative BH3-only indirect activation model [230]. Although the exact mechanism for BH3 mimetics is unclear, the clinical benefits make them attractive antitumor mitochondrial therapies.

## 6. Concluding Remarks

A multitude of biological responses and signaling pathways impacted by altered cellular redox state is a testament to the crucial role that oxidative metabolism plays in cancer cell fate determination. Outstanding contributions over the past couple of decades have clearly highlighted the dichotomy of redox-mediated responses and their effect(s) on myriad processes associated with carcinogenesis and its progression, such as regulation of gene expression, genome instability and mutagenesis, structural and functional modifications of proteins, cell growth and proliferation, cell cycle progression, cellular senescence, and apoptosis execution and resistance. To that end, an intricate crosstalk between altered redox state (and mitochondrial metabolism) and oncogenic or tumor suppressor proteins has been elegantly demonstrated using various model systems. This has seen the emergence of a new paradigm in which cellular redox metabolism appears to dictate cancer cell fate decisions and as such a highly attractive target for novel anticancer drug design and development. The latter include therapeutics to specifically target mitochondrial metabolism, which is a potent source of intracellular ROS. Some of these strategies are already undergoing clinical evaluation such as the Mitocans and MnSOD mimetics. Despite these encouraging developments, there also remain important unresolved issues, such as the design of specific probes for the temporal and spatial detection *in vivo* of various reactive oxygen intermediates, the development of more effective oxidants/anti-oxidants with good therapeutic indices, and the selective delivery of mitochondria-targeted agents. Surely, the intricate interplay between mitochondria and cellular redox state in cancer cell fate is beginning to be realized and exploited for the design of ROS-targeted therapies.

## Figures and Tables

**Table 1:** Summary of the various mitochondrial-related therapeutics in targeting tumor progression.

Category	Drug name	Mechanism	References
Complex I inhibitors	Metformin	Inhibits complex I, disrupting ATP production by OXPHOS.	[170]
	IACS-010759		[171–173]
	OPB-51602		[174, 175]
ATP synthase inhibitors	Oligomycin	Induces heightened superoxide production and apoptosis in tumors.	[178, 179]
	Gboxin	Restricts ATP synthase, increases mitochondria membrane potential.	[180]
Mitochondria biogenesis targeting compounds	Gamitinib	Specifically inhibits tumor mitochondrial HSP90 and induces mitochondria apoptosis.	[182–185]
	Doxycycline	Inhibits mitochondrial biogenesis in bacterial and mammalian cells and reduces mitochondrial translation.	[187, 188]
Inhibitors of mitochondria dynamics	Mitochondrial division inhibitor (e.g., Mdivi 1)	Inhibits mitochondrial fission which prevents cell cycle progression and hence suppresses tumor growth.	[191–194]
SOD mimetics	Manganese porphyrins (e.g., MnTnHex 2 PyP5+)	Mimics MnSOD activity; increases cellular ROS levels, and ultimately induces cell death. Also inhibits cell migration and invasion.	[198, 199]
	Nitroxides (e.g., Mito TEMPOL)	Antioxidative function; induces DNA damage, apoptosis, and mitochondrial distress in tumors.	[200, 201]
Bcl-2 inhibitors	ABT-263 (Navitoclax)	BH3 mimetic that targets antiapoptotic Bcl-2 and Bcl-xL.	[206, 207]
	ABT-737		
	ABT-199 (Venetoclax)	BH3 mimetic that has affinity for antiapoptotic Bcl-2.	[209–213]

## Data Availability

It is a review paper and no data is included.

## Abbreviations

4-HNE: 4-Hydroxynonenal
8-oxo-dG: 8-Oxo-2′-deoxyguanosine
a-KG: Alpha-ketoglutarate
a-KGDH: Alpha ketoglutarate dehydrogenase
ADP: Adenosine diphosphate
Akt: Protein kinase B
ALCL: Anaplastic large cell lymphoma
ALL: Acute lymphocytic leukemia
AML: Acute myeloid leukemia
AMP: Adenosine monophosphate
AMPK: AMP-activated protein kinase
ANT: Adenine nucleotide translocase
ARE: Antioxidant response element
ASK-1: Apoptosis signal-regulating kinase 1
ATP: Adenosine triphosphate
Bak: BCL-2 associated X protein
Bax: BCL-2 homologous antagonist killer
BCL-2: B-cell lymphoma 2
BCL-XL: BCL- extra large
Bcr/Abl: Breakpoint cluster/Abelson
BH3: BCL-2 homology 3 region
Bim: BCL-2-like protein 11
CAFs: Cancer-associated fibroblasts
c-FLIP: CASP8 and FADD-like apoptosis regulator
CKI: Cyclin-dependent kinase inhibitor
CML: Chronic myelogenous leukemia
CoQ: Coenzyme Q
COX: Cyclooxygenase
DDR: DNA damage response
DNA: Deoxyribonucleic acid
dNTPs: Nucleoside triphosphate
ECM: Extracellular matrix
EGFR: Epidermal growth factor receptor
EMT: Epithelial-mesenchymal transition
ERK: Extracellular-signal regulated kinase
ETC: Electron transport chain
FAD: Flavin adenine dinucleotide
FADD: Fas-associated death domain protein
FasL: Fas ligand
FH: Fumarate hydratase
FMN: Flavin mononucleotide
GBM: Glioblastoma
GSH: Glutathione
GTPase: Guanosine trisphosphate hydrolase
HIF-1: Hypoxia-inducible factor 1
HMOX-1: Haem oxygenase 1
IKK: IkB kinase
IL-1: Interleukin 1
IL-6: Interleukin 6
IMS: Intermembrane space
JNK: c-Jun N-terminal kinase
Keap: Kelch-like ECH-associated protein 1
MAPK: Mitogen-activated protein kinase
MMPs: Matrix metalloproteases
MOMP: Mitochondrial outer membrane permeabilization
mPTP: Mitochondrial permeability transition pore
mTOR: Mammalian target of rapamycin
NAD: Nicotinamide adenine dinucleotide
NADPH: Nicotinamide adenine dinucleotide phosphate hydrogen
NF-kB: Nuclear factor kappa-light-chain-enhancer of activated B cells
NOX: NAPH oxidase
Nrf2: Nuclear factor erythroid 2-related factor 2
NSCLC: Nonsmall cell lung cancer
OCT: Organic cation transporter
OIS: Oncogene-induced senescence
OXPHOS: Oxidative phosphorylation
PFK-1: Phosphofructokinase 1
PI3K: Phosphoinositide 3-kinase
PIP_3_: Phosphatidylinositol (3,4,5)-triphosphate
PPP: Pentose phosphate pathway
PTEN: Phosphatase and tensin homolog
PUMA: p53 upregulated modulator of apoptosis
RNA: Ribonucleic acid
ROS: Reactive oxygen species
SASP: Senescence-associated secretory phenotype
SIPS: Stress-induced premature senescence
SOD: Superoxide dismutase
SUV: Suppressor of Var1
tBid: Truncated BH3-interacting domain death agonist
TCA: Tricarboxylic acid cycle
TIGAR: TP53-induced glycolysis and apoptosis regulator
TKI: Tyrosine kinase inhibitor
VDAC: Voltage-dependent anion channel
VEGF: Vascular endothelial growth factor.
